# Sindbis Virus Infection in Non-Blood-Fed Hibernating *Culex pipiens* Mosquitoes in Sweden

**DOI:** 10.3390/v12121441

**Published:** 2020-12-14

**Authors:** Alexander Bergman, Emma Dahl, Åke Lundkvist, Jenny C. Hesson

**Affiliations:** Department of Medical Biochemistry and Microbiology, Zoonosis Science Center, Uppsala University, SE-751 23 Uppsala, Sweden; alexander.brg@outlook.com (A.B.); Emma.dahl@gmail.com (E.D.); ake.lundkvist@imbim.uu.se (Å.L.)

**Keywords:** virus persistence, temperate region, vectors, arbovirus, alphavirus, overwintering, *Culex torrentium*

## Abstract

A crucial, but unresolved question concerning mosquito-borne virus transmission is how these viruses can remain endemic in regions where the transmission is halted for long periods of time, due to mosquito inactivity in, e.g., winter. In northern Europe, Sindbis virus (SINV) (genus alphavirus, *Togaviridae*) is transmitted among birds by *Culex* mosquitoes during the summer, with occasional symptomatic infections occurring in humans. In winter 2018–19, we sampled hibernating *Culex* spp females in a SINV endemic region in Sweden and assessed them individually for SINV infection status, blood-feeding status, and species. The results showed that 35 out of the 767 collected mosquitoes were infected by SINV, i.e., an infection rate of 4.6%. The vast majority of the collected mosquitoes had not previously blood-fed (98.4%) and were of the species *Cx. pipiens* (99.5%). This is the first study of SINV overwintering, and it concludes that SINV can be commonly found in the hibernating *Cx. pipiens* population in an endemic region in Sweden, and that these mosquitoes become infected through other means besides blood-feeding. Further studies on mosquito ecology and viral interactions are needed to elucidate the mechanisms of the persistence of these viruses over winter.

## 1. Introduction

Mosquitoes are vectors of many viruses responsible for causing disease in humans. Despite much research effort, many basic questions on the impact of mosquito ecology on virus transmission remain. One of the most intriguing unresolved questions is how viruses persist over periods of low or non-existing vector activity, such as dry or cold seasons. In temperate regions, this can correspond to months of vector inactivity over winter, which has impact on transmission of viruses such as West Nile virus (WNV), Japanese encephalitis virus (JEV) (*Flaviviridae*), and Sindbis virus (SINV) (genus alphavirus, *Togaviridae*), all utilizing vector mosquitoes belonging to the *Culex* genus.

The suggested mechanisms for viral persistence past adverse periods for the vector include: (a) seasonal re-introduction of virus, (b) persistent infections in vertebrate hosts, and (c) virus infected hibernating mosquitoes [1]. The mechanism that has gathered most scientific evidence is virus persistence in inseminated, hibernating mosquito females. Both JEV and WNV have been found in hibernating females in Asia and North America/Europe, respectively [1,2,3,4,5]. However, reported infection rates are usually low, and most studies showed that the majority of hibernating females had never taken a blood-meal, thus precluding the conventional route of infection trough blood-feeding [1,6,7].

To investigate the mechanisms of viral persistence over winter, we studied SINV in hibernating female *Culex* mosquitoes in an endemic region in Sweden. SINV utilizes passerine birds as its vertebrate host and occasionally infects humans, causing fever, rash, and in some cases long-lasting arthritis [8,9]. It occurs across the Old World, forming six different genotypes [10]. The only genotype known to be associated with human disease is genotype I, which can be found in Europe and Africa. Phylogeographic studies have shown that northern European isolates of SINV have only once been successfully introduced to the region from central Africa in the 1920s and have since remained endemic in the region [11]. Thus, a mechanism of persistence over winter, not involving reintroduction, must exist, but through means still unknown. The aim of this study was to investigate if SINV can persist through winter in the hibernating mosquito population, and whether these females have taken a blood meal prior to hibernation. Our results show that infection rates in the hibernating *Cx. pipiens* population are high, and that the majority of infected females have not taken a blood meal before entering the hibernation site. 

## 2. Materials and Methods

**Figure 1:**
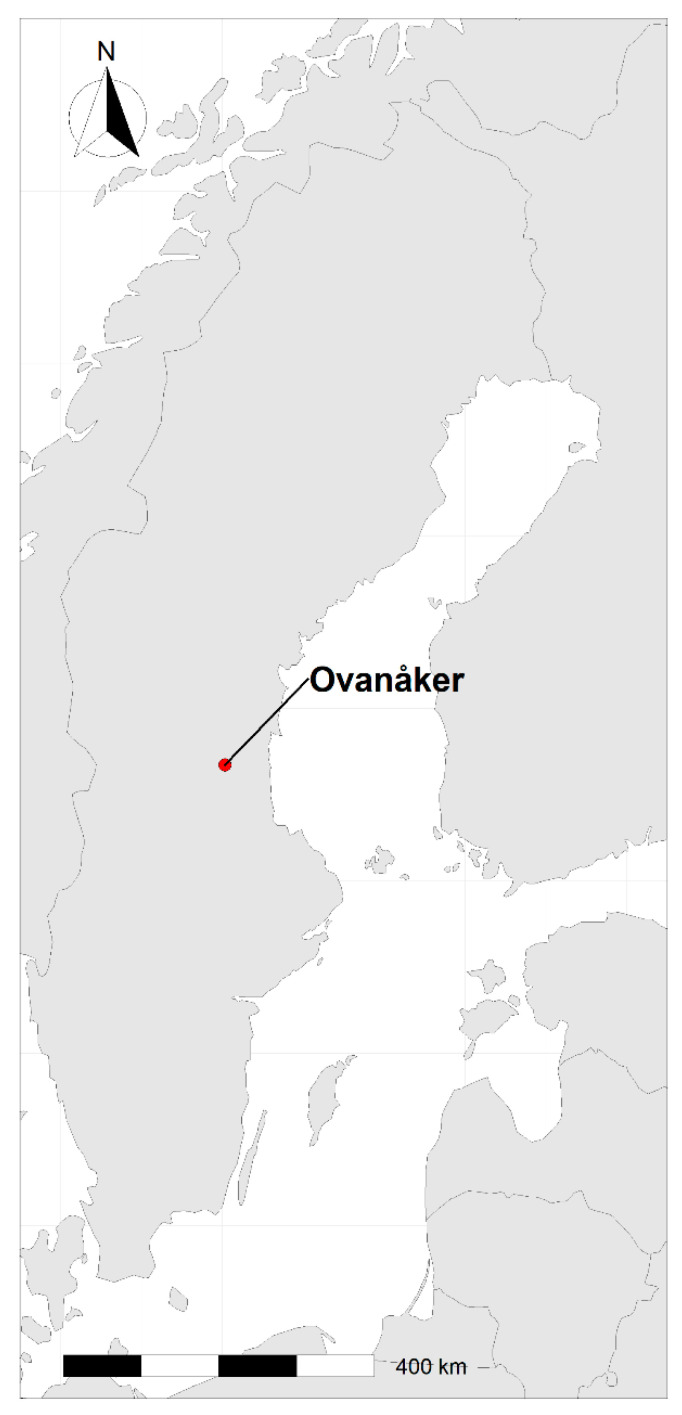
Hibernating *Culex* mosquitoes were collected in the Ovanåkers commune, Sweden, in the winter season 2018–2019.

Hibernating *Culex* mosquito females were collected with aspirators at two occasions for one winter season: on 6–8 December 2018 and 14–16 February 2019. The collections were performed in 14 food storage cellars keeping temperatures just above freezing, in the Ovanåkers commune, Sweden (61.34366, 16.06638) (Figure 1).

**Figure 2:**
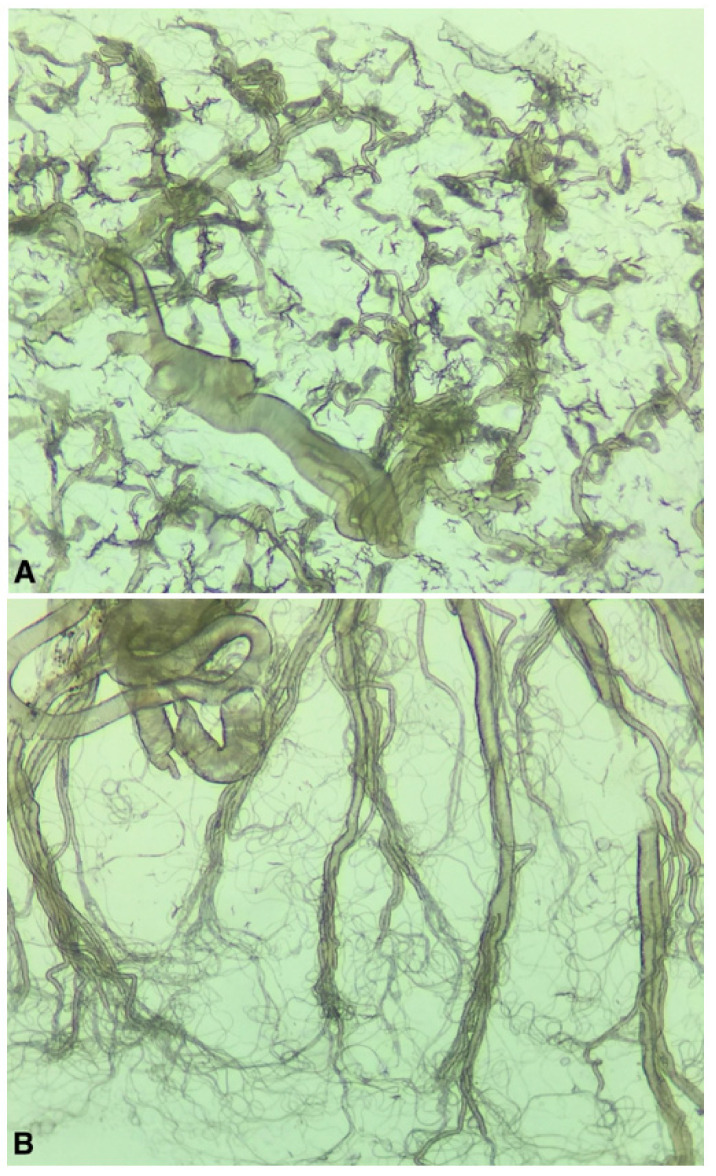
Dissected mosquito ovaries (200× magnification) from a nulliparous female with tightly coiled tracheoles (**A**), and a parous female with uncoiled tracheloes (**B**).

The collected mosquitoes were brought back alive to the laboratory and kept alive for 4–13 days at room temperature and an 18:6 light regime and were given 10% sugar water. *Cx. pipiens pipiens/torrentium* females were then killed by freezing, morphologically identified [12], and freshly dissected for parity, using the tracheal technique [13]. In short, ovaries from each specimen were removed and mounted on a microscope slide for inspection under a microscope. In females that have not blood fed and produced eggs (i.e., are nulliparous) the tracheols are tightly coiled around the ovarioles, resembling skeins (Figure 2A). In females that have previously blood-fed and produced eggs (i.e., are parous) the tracheloes become uncoiled, resembling a thread-like net (Figure 2B). The dissection equipment was washed in ethanol between each sample. The remains of the bodies after dissection were saved at −80 °C and later individually homogenised in 500 μL PBS supplemented with 20% FBS and 2% PenStrep (Thermo Fischer Scientific, Waltham, US). Homogenization was performed by bead beating using two steal beads and a Tissuelyser II™ (Qiagen, Hilden, Germany) at 25 Hz for two minutes.

As an initial screening, 50 μL homogenate from individual mosquitoes were pooled with homogenates from nine other mosquitoes (except for one pool that was made up of seven mosquitoes). From these, 50 μL of homogenate was used for RNA extraction using the RNeasy^®^ Mini Kit (Qiagen, Hilden, Germany). RNA extracts were used as a template in a reverse transcription quantitative PCR (RT-qPCR) using the TaqMan™ RNA-to-C_T_™ 1-step kit (Applied Biosystems, Waltham, MA, US) with primers and probe designed by Jöst et al. [14], targeting 134 base pairs (bp) of the SINV NSP-1 gene (Fwd: 5′-CACWCCAAATGACCATGC-3′, Rev: 5′-KGTGCTCGGAAWACATTC-3′, Probe 5′-FAM-CAGAGCATTTTCGCATCTGGC-BHQ1-3′). All samples were run in duplicates together with two negative controls for each run. Positive controls were not included in any of the RT-qPCR runs together with samples to prevent contamination. 

RNA from individual samples included in pools that were positive by the RT-qPCR were thereafter individually extracted and subjected to RT-qPCR, using the same methods described above. All RT-qPCR positive samples were sent for Sanger sequencing (Macrogen Europe, Amsterdam, the Netherlands) and BLASTed. PCR-positive samples were also analyzed by a second, newly designed conventional PCR (Fwd: 5′-TAGACGTAGACCCTCAGAGTCC-3′, Rev: 5′-CCTTGATCTTCTCATGCAAGTTC-3′) to amplify 336 bp encompassing the 134 bp region of the RT-qPCR. This new conventional PCR was used to obtain a longer product that would be more suitable for sequencing (Macrogen recommends that products should ideally be over 200 bp). Infection rates were calculated as the number of positive individual mosquitoes/the total number of mosquitoes tested x 100. Differences in infection rates between the two collection periods and incubation time were tested for significance using the chi-square test.

Virus isolation attempts were performed on homogenates from all RT-qPCR positive mosquitoes. These were inoculated onto Vero cells that were maintained in DMEM, 5% FBS and 1% Pen Strep (Thermo Fischer Scientific, Waltham, US) at 37 °C, 5% CO_2_, and observed for cytopathic effects for five days.

**Figure 3:**
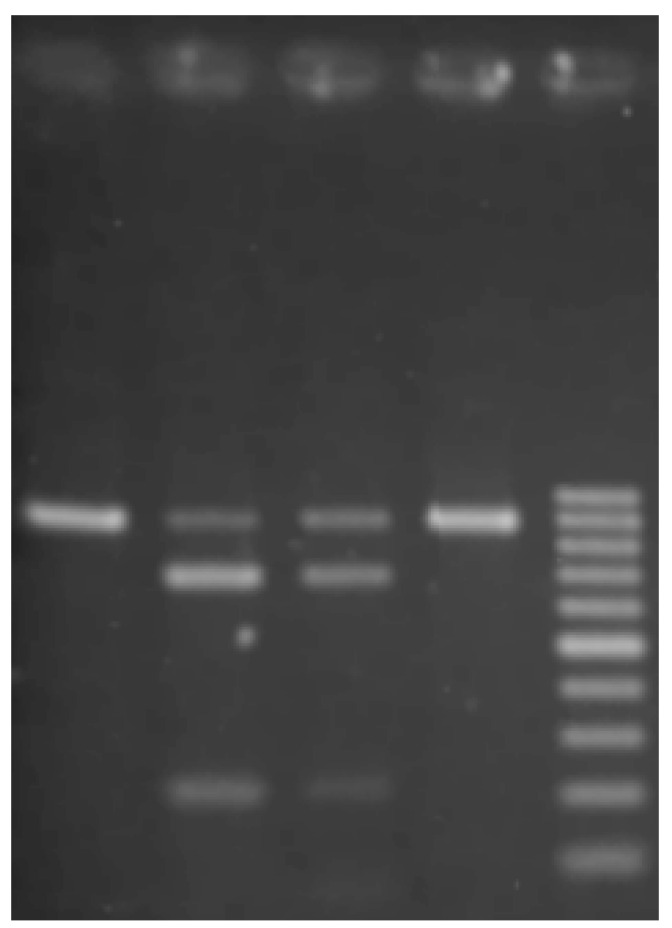
Agarose gel showing the diagnostic bands for identification of *Cx. pipiens* and *Cx. torrentium*. From the left: *Cx. pipiens* with FspBi, *Cx. pipiens* with SspI, *Cx. torrentium* with FspBi, *Cx. torrentium* with SspI, a 100 bp ladder.

Species identification was performed on a subset of samples, using a molecular assay for distinguishing *Cx. pipiens* and *Cx. torrentium*, developed by Hesson et al. (2010) [15]. In short, 5 μL of mosquito homogenate was added to 20 μL of dilution buffer and 0.5 μL of DNA release enzyme from the Phire Tissue Direct PCR kit (Thermo Fischer Scientific, Waltham, MA, US) and incubated at 98 °C for 2 min, before being diluted 10× in water. The dilution was used as a template in a conventional PCR (Fwd: 5′-CAACATTTATTTTGATTTTTTGG-3′, Rev: 5′-TCCAATGCACTAATCTGCCATATTA-3′). Products were then subjected to restriction enzyme digestion by enzymes SspI and FspBi (Thermo Fischer Scientific, Waltham, US), and run on a 1.5% Agarose gel. Species diagnostic bands were visualized by GelRed (VWR, Radnor, PA, US) (Figure 3).

## 3. Results

In total, 767 individual mosquitoes were morphologically identified as *Cx. pipiens/torrentium* females. From these, 480 were collected in December 2018 and 287 in February 2019. Survival rates of mosquitoes brought into the lab were generally very high, and the vast majority of the collected *Culex* mosquitoes could be used for analyses. All samples were initially analyzed for the presence of SINV RNA by an RT-qPCR in 77 pools, out of which 28 were found positive. Individual samples from the 28 positive pools were subsequently analyzed (*n* = 277), and 35 of these individual samples tested positive, i.e., an infection rate of 4.6%. In general, Ct values were high (34–40), and several samples tested positive in only one out of two wells, indicating very low SINV RNA copy numbers. However, they all gave a signal in at least two independent PCR runs. Low viral RNA copy number is also implicated from the unsuccessful attempts of isolating any active virus. Twenty-four individual mosquito samples could also be sequenced and identified as SINV (Supplementary File S1, Table 1). Due to low quality reads on single base level however, no sequences will be reported to a database.

Positive samples were significantly more often collected in December than in February (*p* < 0.05), and all positive samples except two, came from the December collection. The infection rates measured in December and February were 6.9% and 0.7%, respectively. The highest percentages of positive samples were detected after incubation at 10 days or more (*p* < 0.05) (Table 2). No correlation could be seen between incubation time and Ct value.

From the 767 analyzed samples, 641 could be successfully assessed for parity status. Out of these, the vast majority were nulliparous and only 10 individuals (1.6%) were parous. 

Out of the 35 PCR-positive individuals, 27 could successfully be assessed for parity. All except two were nulliparous (Table 1).

Species identification was performed on 404 out of the 767 individuals. The results showed that 99.5% were *Cx. pipiens* and only two individuals were *Cx. torrentium*. All mosquitoes that were PCR-positive for SINV RNA were identified as *Cx. pipiens*, except three that could not be identified to species (Table 1).

**Figure 4:**
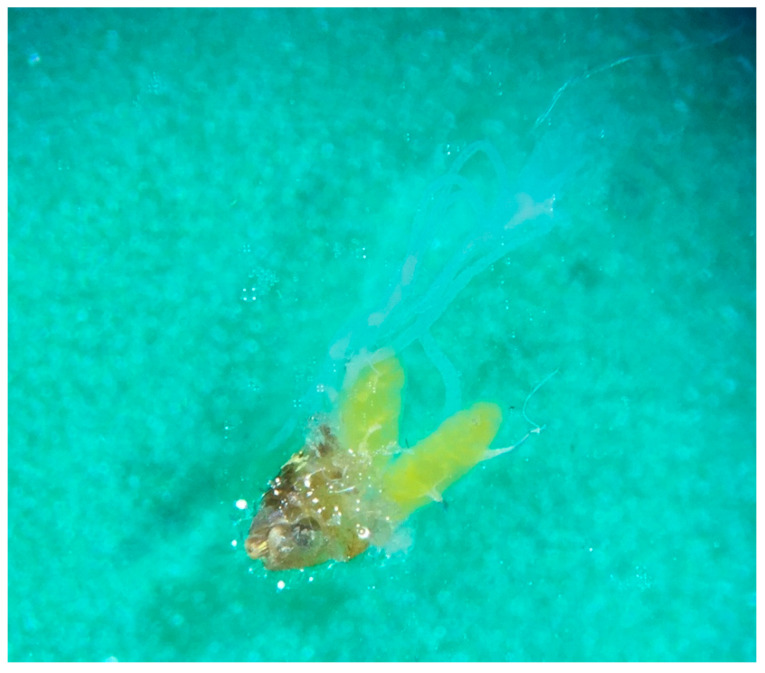
Dissected ovaries in bright yellow colour from an overwintering *Cx. pipiens/torrentium* mosquito collected in winter season 2018–2019.

Three of the uninfected females that could not be assessed for parity had ovaries in a bright yellow colour (Figure 4). In addition to the analyzed *Cx. pipiens/torrentium* mosquitoes, ten *Cx. territans*, and multiple *Anopheles* spp were detected at the hibernation sites.

## 4. Discussion

This study shows, for the first time, that hibernating *Cx. pipiens* in a SINV-endemic region in Sweden is infected by SINV. The studied population had an estimated infection rate of 4.6% in the winter of 2018–2019. This can be compared to the infection rate of 0.8% reported for a late summer population of *Cx. pipiens,* and 3.6% for *Cx. torrentium*, both collected 120 km further south in 2009 [16]. In the late summers of 2001 to 2003, estimates of 1.3%, 1.1%, and 0 infected *Cx. pipiens/torrentium* (not identified to species) were also reported, thus the infection prevalence varies greatly between years [17]. The prevalence of SINV in the hibernating population seems to be as high, or even higher, as in the summer population. This may be a necessity for a virus to successfully utilize vectors for its persistence over winter, as the mortality of hibernating mosquitoes across the winter months could be high and thereby, a large number of infected individuals are needed for enough to survive and start the transmission again in the spring [18]. The mechanism behind the high infection rate is, however, still not known, since most of the females had not blood-fed and thus must have become infected through alternative routes (see below).

The reported infection rates of SINV, both in winter and summer, are exceedingly high in comparison to similar studies focusing on WNV in temperate regions of the US, where infection rates in *Culex* vector spp are usually way below 2% in summer and below 0.1% in winter [2,19]. Interestingly, the infection rate in the present study was significantly lower in February than in December. This may, however, partially also be attributed to that the mosquitoes collected in February were kept alive at room temperature for a shorter period of time. Also, different cellars were sampled at the two occasions; thus, a potential explanation could be that the infected mosquitoes were not evenly distributed between the cellars. This could also be the reason why infection rates differ despite similar incubation time and number of mosquitoes tested in the December sampling (2.3% at 12 days incubation versus 0 at 13 days incubation) (Table 2). All sites visited were only 0.25–4 km apart, so a potential site effect is likely to be on a very local scale.

We also investigated parity status of the hibernating females, and in agreement with other studies we found that the vast majority (98%) of the hibernating females were nulliparous, thus had likely not blood-fed prior to hibernation [1,6,7]. In addition, we could show on an individual level that 25 SINV RNA positive mosquitoes were nulliparous. Thus, the vast majority of SINV infected mosquitoes probably have become infected through other routes than blood-feeding. A potential exception in mosquito physiology that can be demonstrated under laboratory conditions is when a blood-meal is used for energy instead of egg development, referred to as gonotrophic dissociation. This could give the false impression that a female has not blood-fed by assessing her parity status. However, several studies have concluded that this is probably a very rare event in nature [20,21,22,23]. Therefore, we consider it unlikely that gonotrophic dissociation is the underlying explanation to the large number of nulliparous females that were found SINV RNA positive in this study.

Our findings of SINV RNA in nulliparous females raise the important question of how these females were infected. Alternative methods of infection of vectors include, e.g., mechanical, venereal, and vertical transmission from an infected mother to her offspring, with the latter being the most studied for mosquitoes. Experimental studies generally show that vertical transmission of alphaviruses and flaviviruses is very low (1–6‰), however some experimental estimates for WNV show that 8% of adult progeny can be infected [24,25]. The route of infection for SINV in hibernating *Culex* females, including vertical transmission efficiency, remains to be studied.

The lack of virus isolation and the difficulty to obtaining longer sequences is likely a result of the low virus amount in hibernating mosquitoes, observed also by other authors studying WNV and JEV [1,3,24]. Dohm and Turell [26] proposed that keeping mosquitoes in higher temperature for a few days would start replication of WNV, and thus make it easier to detect. We kept our mosquitoes between four and 13 days at room temperature and could indeed see a significant effect on increased detection in mosquitoes that were kept longer than ten days. Despite the prolonged incubation period at room temperature, virus isolation on Vero cells could not be achieved. In the field, adult *Culex* females will appear from their hibernation sites as temperatures rise by the end of March/beginning of April. Their main drive after hibernation is to find carbohydrate energy sources before they can start looking for a blood-meal host. The first *Culex* eggs can usually be found in May/June, and the earliest detection of SINV neutralizing antibodies in birds is in mid-May [9]. Thus, the incubation time of virus in mosquitoes emerging in the spring could be as long as 1–2 months, however, in temperatures well below room temperature. Whether this longer time period (albeit at lower temperatures) is enough to increase virus replication, or whether other stimuli are needed is not known.

The main vector of SINV in Sweden is considered to be *Cx. torrentium*, based on its high field infection rate in summer, its high vector competence in laboratory experiments, and exceed in abundance over *Cx. pipiens* [16,27,28]. In the present study however, we could only find two individuals of *Cx. torrentium*. This is in agreement with previous reports by Jaenson [7] and Service [6] in Sweden and the UK respectively, that few or no hibernating *Cx. torrentum* can be found in man-made shelters. This mosquito species has long been under-studied, shadowed by the much more widespread and morphologically indistinguishable *Cx. pipiens,* but the increasing knowledge of its importance as a vector highlights the need for more studies on the biology of *Cx. torrentium* [29,30].

The climate in SINV endemic regions in Sweden is unsuitable for mosquito activity for long periods of time, often exceeding five months. This study shows that SINV may persist through this season in one of its hibernating *Culex* vectors. Further studies are needed to assess whether other means of persistence, such as persistent infection and recrudescent virema in its bird hosts are also needed to maintain SINV transmission between seasons.

## Figures and Tables

**Table 1:** Detection of SINV RNA in overwintering *Cx. pipiens/torrentium* mosquitoes in Sweden, during the winter season 2018–2019.

Species	Parity Status	Collection Period	Incubation Time (Days)	PCR Positive	Sequencing Positive
*Cx. pipiens*	nulliparous	6 December	5	yes	yes
*Cx. pipiens*	unknown	6 December	5	yes	no
*Cx. pipiens*	unknown	6 December	5	yes	yes
*Cx. pipiens*	nulliparous	7 December	10	yes	yes
*Cx. pipiens*	nulliparous	7 December	10	yes	yes
*Cx. pipiens*	nulliparous	7 December	10	yes	no
*Cx. pipiens*	nulliparous	7 December	10	yes	no
*Cx. pipiens*	nulliparous	7 December	11	yes	yes
*Cx. pipiens*	parous	7 December	11	yes	yes
*unknown*	unknown	7 December	12	yes	no
*Cx. pipiens*	nulliparous	7 December	12	yes	yes
*Cx. pipiens*	nulliparous	7 December	12	yes	yes
*Cx. pipiens*	unknown	7 December	12	yes	yes
*Cx. pipiens*	nulliparous	7 December	12	yes	yes
*Cx. pipiens*	unknown	7 December	12	yes	no
*Cx. pipiens*	nulliparous	7 December	12	yes	no
*Cx. pipiens*	nulliparous	7 December	12	yes	yes
*Cx. pipiens*	nulliparous	7 December	12	yes	no
*unknown*	nulliparous	7 December	12	yes	no
*Cx. pipiens*	nulliparous	7 December	12	yes	yes
*Cx. pipiens*	nulliparous	7 December	12	yes	yes
*Cx. pipiens*	nulliparous	7 December	12	yes	yes
*Cx. pipiens*	unknown	7 December	12	yes	yes
*Cx. pipiens*	nulliparous	7 December	12	yes	no
*Cx. pipiens*	nulliparous	7 December	12	yes	yes
*Cx. pipiens*	parous	7 December	12	yes	yes
*Cx. pipiens*	nulliparous	7 December	12	yes	yes
*Cx. pipiens*	nulliparous	7 December	12	yes	yes
*Cx. pipiens*	nulliparous	7 December	12	yes	yes
*Cx. pipiens*	nulliparous	7 December	12	yes	yes
*Cx. pipiens*	nulliparous	7 December	12	yes	yes
*Cx. pipiens*	nulliparous	7 December	12	yes	yes
*Cx. pipiens*	unknown	7 December	12	yes	yes
*unknown*	unknown	15 February	11	yes	no
*Cx. pipiens*	nulliparous	15 February	11	yes	no

**Table 2:** Incubation times at room temperature and number of *Cx. pipiens/torrentium* mosquitoes analyzed and tested positive for SINV RNA. The mosquitoes were collected at two different time periods and kept alive at room temperature for different numbers of days.

Incubation Time (Days)	Collection Period	Number Analyzed	SINV Positive: Number (Percent)
4	December	10	0 (0)
	February	40	0 (0)
5	December	50	3 (6)
	February	NA	NA
6	December	NA	NA
	February	50	0 (0)
7	December	10	0 (0)
	February	140	0 (0)
8	December	32	0 (0)
	February	NA	NA
10	December	88	4 (4.5)
	February	40	0 (0)
11	December	80	2 (2.5)
	February	17	2 (12)
12	December	110	24 (23)
	February	NA	NA
13	December	100	0 (0)
	February	NA	NA

## Supplementary Materials

The following are available online at https://www.mdpi.com/1999-4915/12/12/1441/s1, File S1. Partial Sindbis virus sequences recovered from hibernating *Culex* females.
