# Supplementary material for: Sindbis Virus Infection in Non-Blood-Fed Hibernating Culex pipiens Mosquitoes in Sweden
Source: Viruses. 2020 Dec 14;12(12):1441. doi: 10.3390/v12121441 (PMC7765111; doi:10.3390/v12121441)
Supplement: Supplementary file 1 [file viruses-12-01441-s001.pdf]

>H18-33
GAGGTAGTAGCACAGCAGGCCACTCCAAATGACCATGCTAATGCCAGAGCATTTTCGCATCT
GGCCAGTAAACTAATCTGACTGGTAGGTTCCTACCACAGCGACGATTTTGGACATAGGCAGC
GCACCGGCTCGTAGAATGTTTTCCGAGCACCAGTATCACTGCGTTTG-
CCCCATGCTTAGTCCAGAAGATCCGGACCGCATGATGAAATATGCCAGCAAACTGGCGGAAA
AAGCATGCAAGATTACAAACAAAAACTTGC

>H18-42
TCTGACTGG-
AGGTTCCTACCACAGCGACGATTTTGGACATAGGCAGCGCACCGGCTCGTAGAATGTTTTCCG
AGCACC

>H18-121
TCTGGCCAGTAAACTAATCTAACTGGTAGGTTCCTACCACAGCGACGATTTTGGACATAGGCA
GCGCACCGGCTCGTAGAATGTTTTCCGAGCAC

>H18-126
TCTGGCAGTAACTAATCTAGCTGGTAGGTTCCTACCACAGCGACGATTTTGGACATAGGCAGC
GCACCGGCTCGTAGAATGTTTTCCGAGCAC

>H18-217
GTAAACTAATCGAGCTGG-
AGGTTCCTACCACAGCGACGATTTTGGACATAGGCAGCGCACCGGCTCGTAGAATGTTTTCCG
AGCACC

>H18-229
ATTTGAGGTAGTAGCACAGCAGGCCACTCCAAATGACCATGCTTATGCCAGAGCATTTTCGCA
TCTGGCCAGT------------------
GGTTCCTACCACAGCGACGATTTTGGACATAGGCAGCGCACCGGCTCGTAGAATGTTTTCCGA
GCA

>H18-330
GAGGTAGTAGCACAGCAGGCCACTCCAAATGACCATGCTAATGCCAGAGCATTTTCGCATCT
GGCCAGTAAACTAATCGAGCTGG-
AGGTTCCTACCACAGCGACGATTTTGGACATAGGCAGCGCACCGGCTCGTAGAATGTTTTCCG
AGCACCAGTATCACTGCGTTTG-
CCCCATGCGTAGTCCAGAAGACCCGGACCGCATGATGAAATATGCCAGCAAACTGGCGGAA
AAAGCATGCAAGATTACAAACAAGAACTTGC

>H18-332
GG-
AGGTTCCTGCCGCACCGACGATGTTGGACATAGGCTGTGCGCCGGGTCGGAAAATGTTTTCCG
AACCCCAGTATCATTGTGTCTG-
CCCCATGCCTAGTCCAGAAAACCCCGACCGCAAGGTAAAATATGCCAACAAACTGGCGGAA
GAAGCGTGCAAGATTTCCAACAAAAACTTGCATGA

>H18-336
ATTTGAGGTAGTAGCACAGCAGGCCACTCCAAATGACCATGCTTATGCCAGAGCATTTTCGCA
TCAGGTCAGTGCTCTAATCGAGCTGG-
AGGTTCCTACCACAGCGACGATTTTGGACATAGGCAGCGCACCGGCTCGTAGAATGTTTTCCG
AGCACCAGTATCACTGCCTTTG-
CCCCATGGTTAGTCCAGAAAATCCCGGCCGCCAGATGAACTATGCCAACAAATTGGGGGAAA
AAC

>H18-337
GG-
AGGTTCCTACCACAGCGACGATTTTGGACATAGGCAGCGCACCGGCTCGTAGAATGTTTTCCG
AGCACC

>H18-340
GTAACTAATCGAGCTGG-
AGGTTCCTACCACAGCGACGATTTTGGACATAGGCAGCGCACCGGCTCGTAGAATGTTTTCAG
AGTTCC

>H18-345
CACAGCGACGATTTTGGACATAGGCAGCGCACCGGCTCGTAGAATGTTTTCCGAGCACC

>H18-346
GAGGTAGTAGCACAGCAGGCCACTCCAAATGACCATGCTAATGCCAGAGCATTTTCGCATCT
GGCCAGTAAACTAATCGAGCTGG-
AGGTTCCTACCACAGCGACGATTTTGGACATAGGCAGCGCACCGGCTCGTAGAATGTTTTCCG
AGCACCAGTATCACTGCGTTTG-
CCCCATGCGTAGTCCAGAAGACCCGGACCGCATGATGAAATATGCCAGCAAACTGGCGGAA
AAAGCATGCAAGATTACAAACAAGAA

>H18-347
AACTAATCGAGCTGG-
AGGTTCCTACCACAGCGACGATTTTGGACATAGGCAGCGCACCGGCTCGTAGAATGTTTTCCG
AGCAC

>H18-348
CTAATCGAGCTGG-
AGGTTCCTACCACAGCGACGATTTTGGACATAGGCAGCGCACCGGCTCGTAGAATGTTTTCCG
AGCAC

>H18-354
GCAGGCCACTTCAACTTACCATGCTAATGCCATAGGATTTTCCCTTCTGGCCAGTAAACTAAT
CGAGCTGG-
AGGTTCCTACCACAGCGACGATTTTGGACATAGGCAGCGCACCGGCTCGTAGAATGTTTTCCG
AGCACCACTATCACCGCGTTTG-
CCCCATGCGTATCCCAGAAGACCCGGACCGCATGATGAAATATGCCACCAAATTGGCGTAAA
AAGCATGTGAGATTACCCAC

>H18-355
CTAATCGAGCTGG-
AGGTTCCTACCACAGCGACGATTTTGGACATAGGCAGCGCACCGGCTCGTAGAATGTTTTCCG
AGCA

>H18-356
GAGCATTTTCGCTTCTGGCCAGTAAACTAATCGAGCTGG-AGGT-
CCTACCACAGCGACGATTTTGGACATAGGCAGCGCACCGGCTCGTAAAATGTTTTCCGAGCA
CCAGTATCACTGCGTTTG-
CCCCATGCGTATTCCAGAAGACCCGGACCGCATGATGAATTATGCGAGCAAACTGGCGGAAA
AAGTTTGCAAGATTACAAACTAGAACTTGCATGAG

>H18-357
GTAACTAATCGAGCTGG-
AGGTTCCTACCACAGCGACGATTTTGGACATAGGCAGCGCACCGGCTCGTAGAATGTTTTCCG
AGCA

>H18-358
CTAATCGAGCTGG-
AGGTTCCTACCACAGCGACTATCTTGGACATAGGCAGCGCACCGGCTCGTAGAATGTTTTCCG
AGCACC

>H18-364
CTAATCGAGCTGGTAGGTTCCTACCACAGCGACGATTTTGGACATAGGCAGCGCACCGGCTC
GTAGAATGTTTTCCGAGCAC

>H18-366
GGTAACTAATCGAGCTGG-
AGGTTCCTACCACAGCGACGATTTTGGACATAGGCAGCGCACCGGCTCGTAGAATGTTTTCCG
AGCACC

>H18-367
AAGAGCTTCCCGCATTTGAGGTAGTAGCACAGCAGGCCACTCCAAATGACCATGCTAATGCC
AGAGCATTTTCGCATCAGGTCAGTAGATCGATAAAACTGG-
AGGTAGCTGCCACGCCGACTATTTTGGACATTCGCTGT-
TGGCGAATCTTCAAATGGTTTCCGATCCCCGCCATCACTGCAAATGCCCCCAGGCTTAGCCCG
GAAATTCGGACCGGCTTGTTGAACTATGCCACCAAATTGCGGAAAAATCTTTGCAAGTTTCCA
AAAAAAACTTGGCATAAG

>H18-368
TCGAGCTGG-
AGGTTCCTACCACAGCGACGATTTTGGACATAGGCAGCGCACCGGCTCGTAGAATGTTTTCCG
AGCA
